# Supplementary figures and images for: The Impact of Primary Tumor Location on Long-Term Oncological Outcomes in Patients with Upper Tract Urothelial Carcinoma Treated with Radical Nephroureterectomy: A Systematic Review and Meta-Analysis
Source: J Pers Med. 2021 Dec 14;11(12):1363. doi: 10.3390/jpm11121363 (PMC8708118; doi:10.3390/jpm11121363)

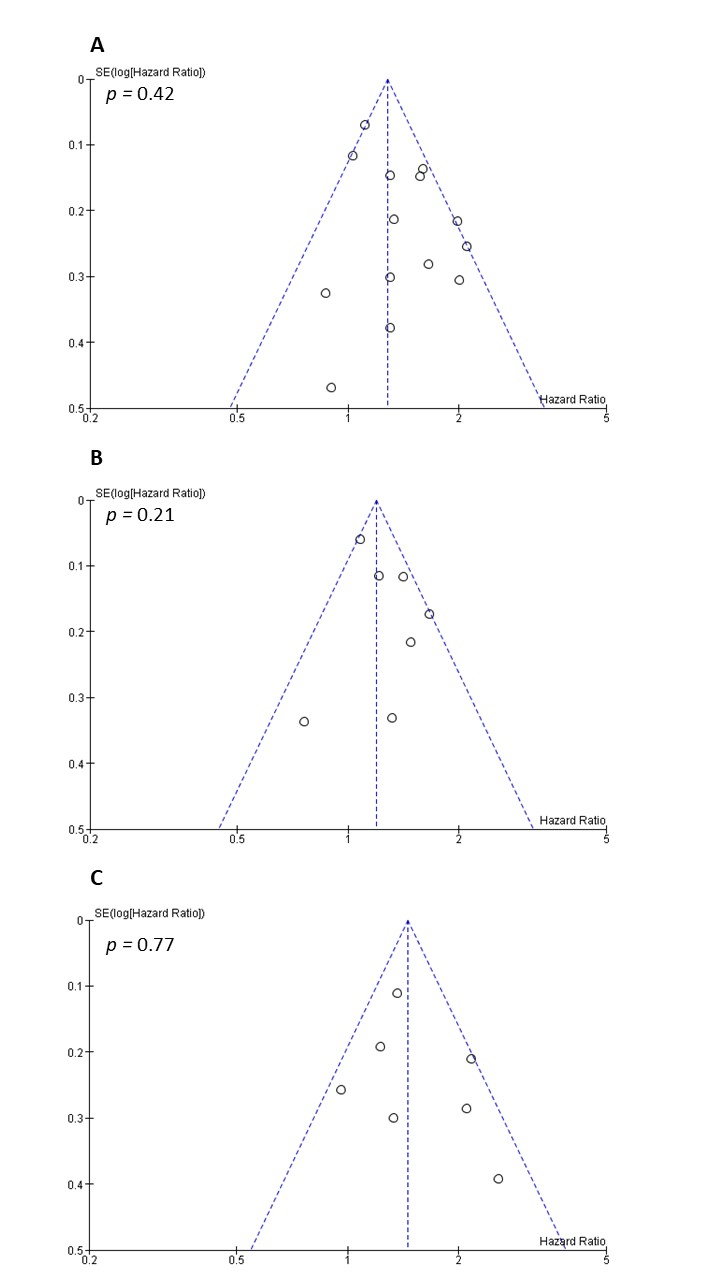

Supplement: Supplementary file 1 [file jpm-11-01363-s001.zip › Supplementary Figure 1.jpg]

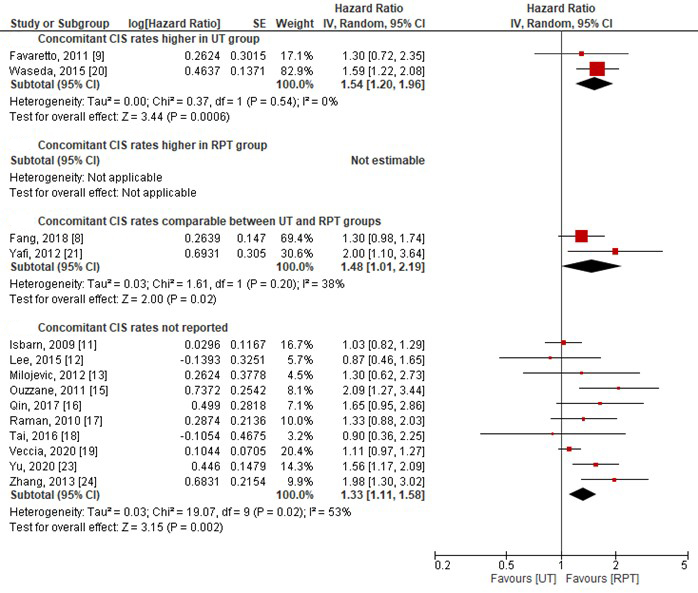

Supplement: Supplementary file 1 [file jpm-11-01363-s001.zip › Supplementary Figure 2.jpg]
